# Frequency, Timing, and Patient Factors Associated with Recurrence of Disseminated Cutaneous Coccidioidomycosis

**DOI:** 10.3390/jof12020120

**Published:** 2026-02-09

**Authors:** Nathan A. Chow, Janis E. Blair

**Affiliations:** 1 Department of Medicine, Mayo Clinic Arizona, Scottsdale, AZ 85259, USA; 2 Mayo Clinic School of Graduate Medical Education, Scottsdale, AZ 85259, USA; 3 Division of Infectious Diseases, Mayo Clinic, Phoenix, AZ 85054, USA

**Keywords:** coccidioidomycosis, cutaneous, dissemination, treatment, recurrence, valley fever

## Abstract

Disseminated cutaneous coccidioidomycosis (DCC) is an uncommon manifestation of *Coccidioides* infection resulting from hematogenous spread to the skin. While recurrence after treatment discontinuation has been reported in 17 to 50 percent of cases, associated frequency, timing, and risk factors are not well defined. We conducted a retrospective review of biopsy-proven or probable DCC cases between January 2008 and March 2024, and investigated for evidence of recurrence. Demographic, clinical, and treatment data were abstracted, including antifungal regimen, adherence, immune status, and coccidioidal titers. A total of 45 subjects met the inclusion criteria, including 27 immunocompetent and 18 immunosuppressed patients. Eleven (24.4%) experienced one or more recurrences, totaling 22 recurrences; 19 of these (86.4%) occurred at previously affected sites. Ten immunocompetent patients (37.0%) had 21 total recurrences, while one immunosuppressed patient (5.6%) experienced a single recurrence. Median antifungal-free interval before recurrence was 14 months (range, 1–96), and 10 recurrences (90.9%) occurred while off antifungal therapy. Ten patients underwent initial surgical excision, with four (40.0%) experiencing a total of 11 recurrences afterwards. DCC recurrence was common, mostly among immunocompetent individuals not on suppressive antifungal therapy, and frequently presented with multiple recurrences. Recurrences were almost always at prior lesion sites, often years after treatment discontinuation.

## 1. Introduction

Coccidioidomycosis, commonly referred to as Valley fever, is a fungal infection caused by *Coccidioides* spp., which are endemic to restricted geographic regions such as the southwestern United States (US). While up to two-thirds of infections are subclinical, the most common disease manifestation is a self-limited pneumonia, though extrapulmonary disease can occur in up to 30–50 percent of immunosuppressed patients [1]. In 2019, it is estimated that up to 360,000 symptomatic cases occurred in the US, leading to as many as 28,000 hospitalizations, over ten times higher than public reports, which themselves have increased over threefold since 2000 [2,3]. Climate change models predict an expansion in the geographic distribution of Valley fever over the coming decades, underscoring its growing relevance to public health [4,5].

Whereas primary cutaneous coccidioidomycosis results from direct inoculation of the fungus into broken skin, disseminated cutaneous coccidioidomycosis (DCC) arises from hematogenous spread of *Coccidioides* to the skin and may reflect more severe systemic infection or underlying immune dysfunction [6]. Extrapulmonary infection occurs in ≤1% of individuals with coccidioidal infection [1], and among these, cutaneous dissemination is estimated in 15% to 67% of cases [7,8]. However, few studies describe the timing or frequency of DCC recurrence. Small case series reports recurrence rates after discontinuation of medication ranging from 17% to 50%, but often fail to distinguish cutaneous dissemination from involvement of other soft tissues and are limited by small sample size [8,9,10]. In this study, we aimed to characterize the frequency, timing, and risk factors associated with relapsed DCC in a larger patient population.

## 2. Materials and Methods

We obtained approval from our Institutional Review Board prior to initiation of data collection. Data were drawn from three separate campuses of an academic medical center, including Rochester, MN, USA; Jacksonville, FL, USA; and Phoenix, AZ, USA. Potential DCC cases were identified using a search for International Classification of Diseases (ICD) codes from the 9th and 10th revisions (ICD-9, ICD-10), specifically 114.1–114.3, and 114.9, as well as 38.3, 38.7, and 38.9. We additionally searched institutional pathology reports using the Boolean keyword query “(skin OR cutaneous OR dermal OR *dermis) AND (cocci* OR coccidioidomycosis OR valley fever OR spherule*)”. We searched for patients between 1 January 2008 and 31 March 2024. We conducted a retrospective chart review to identify patients with cutaneous coccidioidal infection, and abstracted the following information from the charts: demographic descriptions, comorbid illnesses and medications (with an assessment of immunity) at the time of coccidioidomycosis diagnosis, and details of initial and subsequent coccidioidal illness (symptoms, laboratory results [microbiology, serology, pathology], details of antifungal treatments, and response to treatment and follow up course). From the notes of the medical provider, we recorded any notation of antifungal medication adherence, if present. We identified immunosuppressed patients as those with active or recent therapies, congenital conditions, or malignancies known to cause moderate-to-severe deficiencies in cellular immunity.

We included patients with biopsy-proven or probable DCC. Biopsy-proven DCC was defined as having the presence of spherules on pathology or the growth of *Coccidioides* on cultures taken from the affected site. In the absence of biopsy-proven coccidioidal skin lesions, we defined probable DCC as cases meeting the following criteria: (1) compatible skin lesions (discrete or clusters of papules, nodules, or plaques, with or without ulcerations) [11], (2) positive coccidioidal serology, (3) appropriate response to antifungal therapy, and (4) absence of a more likely alternate diagnosis. We excluded patients with a history of direct cutaneous inoculation with resultant primary cutaneous coccidioidomycosis and excluded those whose cutaneous involvement was limited to reactive rashes.

We defined recurrence as the reappearance of cutaneous lesions, with relapse more narrowly defined as the reappearance of DCC while still on active antifungal treatment. For each recurrence or relapse, we recorded the total number of episodes, timing, and lesion location. We used simple summary statistics to describe our patient cohort and compared those with and without recurrence or relapse using chi-square or Fisher’s exact test as appropriate; statistical significance was set at *p* < 0.05.

## 3. Results

**Figure 1:**
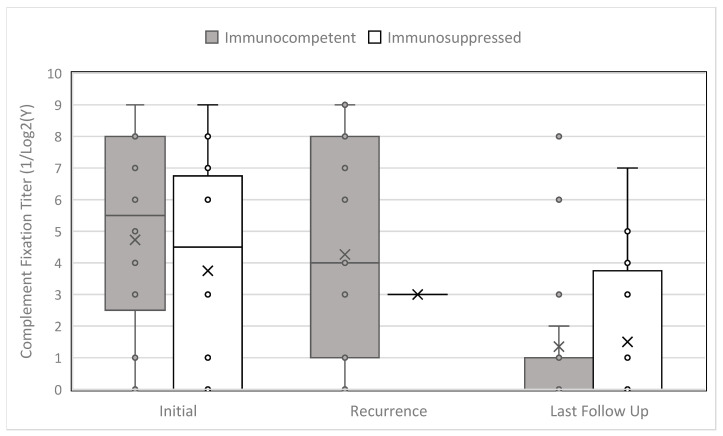
Complement fixation titers at diagnosis, recurrence, and last follow-up, stratified by immune status.

We initially identified 159 potential cases and excluded 114 patients who did not meet the inclusion and exclusion criteria. Our final cohort consisted of 41 biopsy-proven and 4 probable cases of DCC for a total of 45 patients, including 27 immunocompetent and 18 immunocompromised patients. Table 1 summarizes demographic, clinical, treatment, and follow-up characteristics of these 45 patients, and Figure 1 demonstrates median *Coccidioides* complement fixation (CF) titers at diagnosis, recurrence, and last follow-up. The majority of patients were male, white, non-Hispanic, and immunocompetent. Two patients were diagnosed and treated in Rochester, MN, one patient in Jacksonville, FL, and the remainder in Arizona.

Median follow-up duration was 68 months (range 0–336). Eleven patients (24.4%) experienced one or more recurrences, and six patients (13.3%) had two or more. Of 22 total recurrences, 19 (86.4%) occurred at previously affected skin sites. There were no apparent differences in recurrence by race or diabetes status. No significant association between initial CF titer and risk of recurrence was found. Cumulative recurrences by patient characteristics are shown in Table 2.

Four patients relapsed while on antifungal therapy. Suboptimal adherence was documented in two patients, while a third patient had therapeutic fluconazole levels but ultimately responded to an increased daily fluconazole dosage from 400 to 600 milligrams. The fourth patient had self-discontinued his antifungal treatment for approximately one month, but the documentation was unclear whether the pause occurred before or after the recurrence. Ten patients underwent surgical excision of the cutaneous coccidioidal lesions, of whom four (40.0%) experienced a total of 11 recurrences after attempted excision.

The median antifungal-free interval prior to recurrence was 14 months (range, 1–96). Recurrence was noted in 11 (37.9%) non-immunosuppressed patients and one (5.6%) immunosuppressed patient (*p* = 0.014). Median time to recurrence was 16 months after discontinuation of antifungal treatment among immunocompetent versus 35 months among immunosuppressed, with a median treatment duration of 16 (IQR 12 to 36) versus 30 months (IQR 13 to 63), respectively. No recurrence was documented in patients treated for more than 30 months. Among 18 immunosuppressed patients, 13 (72.2%) were treated with long-term (indefinite) suppressive antifungal treatment. The single recurrence noted in the immunosuppressed group occurred while antifungal therapy was paused.

Five-year mortality in the immunosuppressed group was 16.7% (three patients) compared with 6.9% (two patients) in the non-immunosuppressed group; two deaths among the immunosuppressed group were attributed to disseminated coccidioidomycosis; information on the cause of death for the third patient was unavailable. Neither death in the non-immunosuppressed group was attributed to DCC.

## 4. Discussion

This study provides a detailed characterization of disseminated cutaneous coccidioidomycosis (DCC) recurrence in a modern cohort, with a larger sample size than previously available in the literature. Recurrence occurred in approximately one-quarter of patients, with half of those experiencing multiple recurrences. Most recurrences (86.4%) occurred at the same site as prior infection, suggesting that *Coccidioides* may persist in local tissue niches and re-emerge when immune or treatment conditions change. This site-specificity is consistent with patterns seen in other chronic granulomatous infections [12,13] and raises the possibility of localized immune evasion or incomplete tissue clearance [6].

The timing of recurrence varied, with some occurring within months of discontinuation of the antifungal treatment and others occurring years after apparent remission, highlighting the long-term potential for reactivation. That one patient relapsed despite therapeutic fluconazole levels, yet responded to a dosage increase, suggests that there may have been a relatively resistant organism, and that current fungistatic treatments may suppress—but not eradicate—the infection. The lack of statistically significant association between initial CF titers and risk or presence of recurrence may be due to a lack of sample size, diagnostic delays, and the limited ability of titers to discriminate levels of disease burden. These findings emphasize the need for long-term surveillance, regardless of treatment status. Furthermore, our data raise questions as to when, if ever, patients on long-term antifungal therapy without disease activity can be considered cured, as recurrence was noted up to several years after antifungal discontinuation in patients treated long-term with antifungals.

One recurrence occurred among immunocompromised patients, likely reflecting the longer median antifungal treatment durations in this group (35 vs. 16 months in immunocompetent patients) and ongoing antifungal suppression in most patients. This suggests the possibility that prolonged antifungal therapy and prophylaxis may be more influential than immune status alone in preventing recurrence. Recurrence neither correlated with serologic complement fixation titers at discontinuation of antifungal treatment, nor with the absolute peak complement fixation titer. Similarly, no associations were found with race or diabetes status, although this study was likely underpowered to detect small subgroup differences. Medication nonadherence appeared to have contributed to several relapses, emphasizing the need for patient education, adherence, and prolonged follow-up.

Current treatment guidelines of the Infectious Diseases Society of America for DCC recommend initiation of azole therapy with continuation for at least 6–12 months in immunocompetent hosts; our data suggest it may be appropriate to extend this treatment duration if tolerated, especially if any prior recurrence indicates a higher risk for future relapse. Explicit guidelines as to how long suppressive antifungals should be continued in non-meningeal DCC after a patient’s immunosuppression resolves are not currently established; however, for patients with cellular immunodeficiency, we recommend that suppressive therapy be considered while immunosuppression remains, and our data suggest that this may be an effective means of preventing recurrence. Surgical excision of recurrent coccidioidal skin lesions has been suggested to be an effective strategy for ultimate control of DCC [1,6]; our data suggest that DCC may be resilient to excision, though it is difficult to assess whether an excision achieved sufficient margins by chart review.

We acknowledge several limitations to this study, which may limit generalizability. This is a retrospective chart review at a single tertiary referral center, which may introduce selection bias. The study population was predominantly white and did not fully represent all populations. Pediatric patients were not represented. We attempted to characterize medication adherence, but adherence is difficult to assess retrospectively and prone to subjectivity; many patients on antifungals do not routinely undergo therapeutic drug monitoring. The definition of recurrence, meant to avoid false positives, carries a risk of undercounting, especially when involving a site different than that of the original occurrence. In addition, immune status can be dynamic, with many immunosuppressed patients becoming immunocompetent (or vice versa) throughout the observation period, which can affect the clinical significance of recurrence. While a high rate of recurrence was noted in patients who underwent initial surgical excision, conclusions cannot be drawn as to significance, given the small sample size and unclear documentation of surgical margins and depth of excision. Finally, high mortality in the immunocompromised cohort limited the availability of long-term recurrence data.

In summary, we found that recurrence of DCC was common and remained closer to the lower end of the previously estimated rate of 17–50% [5,6]; however, patients remain vulnerable to recurrence even after many years of antifungal therapy. Our findings also further characterize the factors associated with recurrence. Future work should explore how the use of novel treatments such as olorofim, fosmanogepix, and ibrexafungerp—all with potential future applications in *Coccidioides* infections—as well as optimized treatment durations and novel biomarkers, can improve long-term outcomes and help prevent relapse in high-risk patients [14,15,16].

## Figures and Tables

**Table 1:** Summary of characteristics of 45 patients with disseminated cutaneous coccidioidomycosis.

	Immunocompetent, *n* = 27 (%) *	Immunosuppressed, *n* = 18 (%) *
Age, years, mean, (range)	45, (24–74)	59, (25–82)
Male sex	21 (77.8)	12 (66.7)
Race		
White	16 (59.3)	16 (88.9)
Black	7 (25.9)	1 (5.6)
Asian	1 (3.7)	1 (5.6)
Native American	1 (3.7)	0
Declined/Other	1 (3.7)	0
Hispanic Ethnicity	1 (3.7)	0 (0)
Selected Comorbidities		
Diabetes Mellitus	5 (18.5)	0 (0)
Immunosuppression		
Solid organ transplant		6 (33.3)
Bone marrow transplant		1 (5.6)
Hematologic malignancy/deficiency		6 (33.3)
Chemotherapy		2 (11.1)
Disease-modifying rheumatologic agents		5 (27.8)
Chronic steroid use		4 (22.2)
Primary immunodeficiency		1 (5.6)
Other		2 (11.1)
Other locations of *Coccidioides* infections		
None identified	19 (70.4)	8 (44.4)
Coccidioidal pneumonia	8 (29.6)	5 (27.8)
Osteomyelitis		3 (16.7)
Peritonitis		1 (5.6)
Prostatitis		1 (5.6)
Pharyngitis		1 (5.6)
Epidural abscess		1 (5.6)
Diffuse miliary disease		1 (5.6)
Initial DCC locations		
Head and neck	20 (55.6)	10 (41.7)
Torso excluding breast	7 (19.4)	5 (20.8)
Arms	5 (13.9)	4 (16.7)
Legs	4 (11.1)	4 (16.7)
Breast	0	1 (4.2)
Mean duration of follow-up, months (range)	70 (0–336)	35.5 (0–130)
*Coccidioides* CF titer at DCC diagnosis, median (range) ^1^	1:48 (0–1:512)	1:36 (0–1:512)
*Coccidioides* CF titer at first antifungal discontinuation, median (range) ^2^	0 (0–1:8)	0 (0)
*Coccidioides* CF titer at last follow-up, median (range) ^3^	0 (0–1:256)	0 (0–1:128)
Initial antifungal treatment		
Fluconazole	21 (77.8)	13 (72.2)
Posaconazole	1 (3.7)	1 (5.6)
Itraconazole	3 (11.1)	0 (0)
Voriconazole	1 (3.7)	1 (5.6)
Isavuconazole	0 (0)	1 (5.6)
Amphotericin	1 (3.7)	2 (11.2)
Subsequent antifungal treatments ^4^		
Fluconazole	1 (16.7)	1 (16.7)
Posaconazole	0 (0)	3 (50.0)
Itraconazole	5 (83.3)	1 (16.7)
Voriconazole	0 (0)	2 (33.3)
Prescribed indefinite antifungal treatment	6 (22.2)	13 (72.2)
Total patients experiencing recurrence	10 (37.0)	1 (5.6)
Total patients experiencing relapse	4 (14.8)	0 (0)
Number of recurrences after stopping antifungal treatment, per affected patient		
1	4 (40.0)	1 (100)
2	2 (20.0)	
3	3 (30.0)	
≥4	1 (10.0)	
Duration continuous antifungal treatment prior to recurrence, median in months, (range)	11 (2–59)	33 **
Recurrence timing after antifungal discontinuation		
<6 months	5 (33.3)	1 (100)
6–12 months	2 (13.3)	
1–2 years	2 (13.3)	
3–4 years	1 (6.7)	
>5 years	5 (33.3)	
Recurrence at original location	19 (90.5)	1 (100)
Recurrence at new location	2 (9.5)	0
Initial surgical excision	10 (37.0)	0
Recurrences after surgical excision, per affected patient		
1	1 (25.0)	
2	0 (0)	
3	2 (50.0)	
≥4	1 (25.0)	
Shave biopsy	0	3 (16.7)
Recurrence after shave biopsy	0	0
Incision & drainage	2 (7.4)	0
Recurrence after incision & drainage	0	

* unless otherwise specified, ** *n* = 1, no range, ^1^ *n* = 22 for immunocompetent, *n* = 16 for immunosuppressed, ^2^ *n* = 15 for immunocompetent, *n* = 3 for immunosuppressed, ^3^ *n* = 24 for immunocompetent, *n* = 16 for immunosuppressed, ^4^ *n* = 6 for both groups; includes all subsequent antifungals regardless of recurrence.

**Table 2:** Cumulative recurrences over time by patient characteristics.

	Cumulative Recurrences						
	<1 Month	1–3 Months	3–6 Months	1 Year	3 Years	5 Years	10 Years
Characteristic	(# of Active Patients at End of Period)						
Male (*n* = 33)	0 (30)	1 (30)	1 (29)	5 (29)	9 (20)	12 (20)	16 (13)
Female (*n* = 12)	0 (12)	1 (12)	1 (12)	1 (11)	3 (10)	4 (5)	2 (3)
Diabetes (*n* = 5)	0 (5)	1 (5)	1 (5)	1 (5)	2 (5)	2 (5)	4 (3)
Immunocompetent (*n* = 27)	0 (27)	3 (27)	3 (27)	7 (27)	12 (21)	17 (18)	21 (11)
Immunosuppressed (*n* = 18)	0 (17)	0 (17)	0 (16)	0 (15)	1 (9)	1 (6)	1 (3)

## Data Availability

Data will be kept available on request due to privacy restrictions.

## Abbreviations

The following abbreviations are used in this manuscript:

CF	Complement Fixation
DCC	Disseminated Cutaneous Coccidioidomycosis
ICD	International Classification of Diseases
US	United States
